# A Woman Swallows a Razor

**Published:** 1893-01

**Authors:** 


A WOMAN SWALLOWS A RAZOR.—Jane Savage, a working woman in Lincoln, Eng., while cleaning house last week, placed her brother's small razor between her teeth that she might have both hands free to take down a shelf. As she reached for the shelf the razor slipped half way down her throat. She tried to catch her breath, and thus drew the razor so far down that when a surgeon was called he was unable to relieve her. She was taken to the Lincoln Hospital and examined. Her throat was badly swollen, but the razor was gone. Yesterday she complained of severe pains in her stomach, and symptoms of acute inflammation were discovered. This morning, therefore, her stomach were opened. The razor was found, tightly closed as when she swallowed it. The removal of the razor was followed this evening by the relief of all pain. The woman is doing well, and probably will have recovered fully in three weeks.—NEWS from London.

